# In-Situ Real-Time Focus Detection during Laser Processing Using Double-Hole Masks and Advanced Image Sensor Software

**DOI:** 10.3390/s17071540

**Published:** 2017-07-01

**Authors:** Binh Xuan Cao, Phuong Le Hoang, Sanghoon Ahn, Jeng-o Kim, Heeshin Kang, Jiwhan Noh

**Affiliations:** 1 Department of Laser and Electron Beam Application, Korea Institute of Machinery & Materials (KIMM), Daejeon 34103, Korea; xuanbinh.cao@gmail.com (B.X.C.); Shahn@kimm.re.kr (S.A.); jokim@kimm.re.kr (J.K.); khs@kimm.re.kr (H.K.); 2 Department of Nano-Mechatronics, Korea University of Science and Technology (UST), Daejeon 34113, Korea; 3 Department of Material Science and Engineering, Korea Advanced Institute of Science and Technology (KAIST), Daejeon 34141, Korea; hoanglephuong93@kaist.ac.kr

**Keywords:** focal-position detection, laser micromachining, double-hole masks, image sensor software

## Abstract

In modern high-intensity ultrafast laser processing, detecting the focal position of the working laser beam, at which the intensity is the highest and the beam diameter is the lowest, and immediately locating the target sample at that point are challenging tasks. A system that allows in-situ real-time focus determination and fabrication using a high-power laser has been in high demand among both engineers and scientists. Conventional techniques require the complicated mathematical theory of wave optics, employing interference as well as diffraction phenomena to detect the focal position; however, these methods are ineffective and expensive for industrial application. Moreover, these techniques could not perform detection and fabrication simultaneously. In this paper, we propose an optical design capable of detecting the focal point and fabricating complex patterns on a planar sample surface simultaneously. In-situ real-time focus detection is performed using a bandpass filter, which only allows for the detection of laser transmission. The technique enables rapid, non-destructive, and precise detection of the focal point. Furthermore, it is sufficiently simple for application in both science and industry for mass production, and it is expected to contribute to the next generation of laser equipment, which can be used to fabricate micro-patterns with high complexity.

## 1. Introduction

Recently, ultrafast laser processing has become increasingly significant in academic research and engineering. With the capability of fabricating nano-scale 3D patterns with high resolution, laser processing has an enormous number of applications in both science and industry, such as nano-optics, nano-photonics, plasmonics, nano-electronics, and nano-magnetism. However, because of its superfast working, laser processing has a limitation concerning the focusing condition. A poor focusing condition can lead to the permanent destruction of the target sample, a reduction of resolution, a decrease in sensitivity of optic and electronic nano-devices, and low productivity. Precisely adjusting the target sample at the laser focal position, at which the highest optical energy and smallest beam-spot size are acquired, is extremely significant. Therefore, a precise, flexible, and rapid focus detection system is in considerable demand for increasing the productivity as well as accuracy of laser processing.

Several studies that optimize the method of focal position detection and propose a new auto focusing system for laser processing have been reported. Most of them employ the leading technologies and advanced principles of optics, such as confocal microscopy [1,2]; investigation of the optical characteristics of a working laser beam [3,4,5,6,7,8,9], in which the analysis of chromatic aberration of the fabrication beam during laser drilling [3] and laser welding [4,5], the construction of digital holograms [6], reflected-light microscopy [7], integral sliding mode control [8], and confocal point sensor [9] was performed; Fourier fringe analysis [10]; and utilization of the printed focus pattern [11]. Those methods are all brilliant, but they seem to be expensive and not reproducible when applied in the industry with an enormous number of target samples. More importantly, some other studies have already improved the optical setup to explore the focus on a non-planar sample based on diffractive beam samplers [12,13] or the macro/micro dual-drive principle [14]. Furthermore, many auto-focusing devices have been developed for laser direct writing [14,15,16]; laser ablation [17]; automatic microscopy and measurement [18]; laser material processing [19]; two-photon photo-polymerization (TPP)-based micro-fabrication [20]; and several other applications [21,22,23,24,25,26,27,28,29,30] in Shack-Hartmann wave-front sensor fabrication [21], parallel data processing [22], photonic force microscopy [23], controllable mirror-lens retrofocus objective [24], tunable lens focal offset measurement [25], laser micromachining [26], remote sensing [27], automated optical inspection [28], auto-focusing infinity corrected microscopes [29], and direct imaging technology [30] with detailed theoretical models. However, these focus detection systems are complicated, expensive, and inadequate for the mass production of 3D patterns [21,22,23,24,25,26,27,28,29,30]. Moreover, most of them cannot perform the in-situ real-time detection of the focal point during the working period, in general, and during laser fabrication, in particular. The need for a system that can rapidly and precisely detect the focus during laser fabrication is quite urgent in both science and industry because many engineers and scientists wish to increase the reproducibility, speed, and accuracy of laser micromachining. For dealing with this task, the detection system should employ some optical elements that can exceptionally manipulate the detection beam and fabrication beam in proper directions such that the detector should not be damaged by the fabrication beam and the defocusing offset should be well-diagnosed.

In this paper, we propose a new system for in-situ real-time exploration of focal position during laser processing with double-hole masks. The proposed system has higher accuracy, higher reproducibility, and higher speed compared to conventional systems. First, the automatic positioning of the target sample at the focus immediately when the laser fabrication is performed results in high speed. Secondly, owing to high-speed focus detection, the system can fabricate a large number of samples continuously by replacing a sample without resetting the system; therefore, the system can show high reproducibility in the industry. Thirdly, because our image sensor can precisely read the beam-spot separation as well as beam-spot size during processing, the method has high accuracy. The calibration step for fixing the positions of essential optical elements as well as making a complete optical box functioning as a detection system is specifically explained in detail. The detection and fabrication procedures and results, including the linear relation between beam-spot spacing and objective lens-specimen distance and images of laser-drilled holes, are also presented with experiments. The experimental results are consistent with the theory. During the experiment, the image resolution is defined and evaluated based on the results according to distances between optical elements to optimize their positions. A bandpass filter is employed to prevent the fabrication laser from entering the image sensor; thus, the system can detect the focal position and perform fabrication on the target sample simultaneously, which could not be achieved by previous studies. More importantly, the bandpass filter can also adjust the beam-spot images on the image sensor by reducing the speckle patterns induced by the roughness of the target surface. The new image sensor also contributes to the high-precision reading of beam-spot spacing. Furthermore, the focusing and processing steps conducted simultaneously allows for the mass production of laser 3D micro-patterning. The remainder of this paper is organized as follows. First, we analyze the working principle for the focus determination system based on the mathematical model of geometrical optics and derive the formula of measurement resolution. Second, we present the experimental method, including the optical design for simultaneous focus detection and laser fabrication, and calibration steps. Third, the experimental results and discussion are presented. Finally, we draw conclusions on the proposed technique of real-time focus detection.

## 2. Working Principle of the Focus Determination System

**Figure 1:**
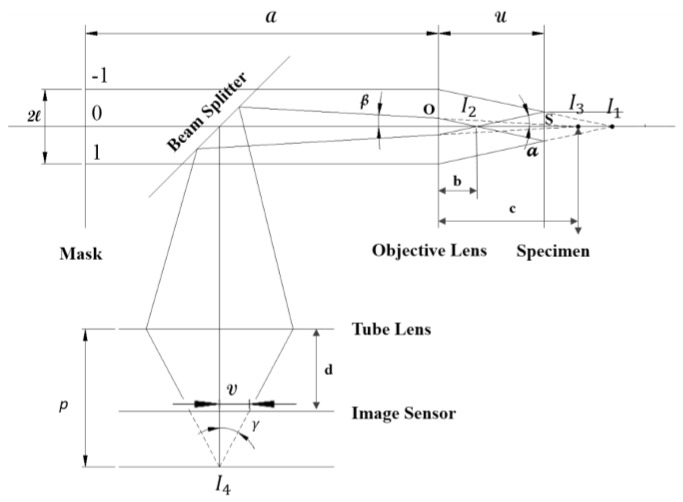
Optical path of the laser beam through several holes at positions −1, 0, and +1, as well as the optical setup.

Figure 1 shows the optical path of the laser beam through several holes at positions −1, 0, and +1, which are at a distance l from each other, and the optical setup of focus detection. In this figure, a is the distance between the mask and the objective lens, b is the distance between $I_{2}$ and the objective lens, c is the distance between $I_{3}$ and the objective lens, d is the distance between the image sensor and the tube lens, f is the focal length of the objective lens, $f^{\prime }$ is the focal length of the tube lens, ρ is the sum of the image sensor–beam splitter distance and beam splitter–objective lens distance, u is the distance between the objective lens and specimen, Δu is the defocusing displacement of the specimen, v is the distance between beam spots induced from two holes at positions 0 and −1 (or +1) on the image sensor, Δv is the change in distance between the beam spots on the image sensor, α is the angle between the laser beam and optical axis after the first transmission through the objective lens, β is the angle between the laser beam and optical axis after the second transmission through the objective lens (reflected beam), γ is the angle between the laser beam and optical axis after a single transmission, and p is the distance between the focusing point of the beams and the tube lens. The holes convert the laser beam from the laser diode into three parallel beams. Subsequently, three parallel beams are reflected on the specimen surface directed to the image sensor, creating two beam dots on the screen. The beam dots are expected to be overlapped when the specimen surface is located at the focal point, i.e., the beam-dot spacing becomes nearly zero, and this signal eventually indicates the focal point. In principle, when the specimen is briefly shifted around the focal position, two beam dots originating from holes −1 and +1 move apart from each other simultaneously, while the beam dot originating from hole 0 remains stable. For equivalence, two double-hole masks are used for detection. One mask contains two holes at positions 0 and −1 (or +1), and another contains two holes at positions −1 and +1. In this section, the mathematical relation between the beam-dot spacing, which is generated from the mask, and the objective lens–specimen distance, as well as the relation between the beam-dot spacing and the defocusing displacement of the specimen are introduced for two situations of the double-hole mask.

According to Figure 1 and the description of beam path above, the intersection at point $I_{2}$ and the intersection at point $I_{1}$ are symmetric over specimen S as a perfect mirror. Thus, we acquire the following as the distance between $I_{2}$ and the objective lens:

(1) b=2u−f.

Subsequently, the intersection at point $I_{3}$ is regarded as the image of the intersection at point $I_{2}$ through the objective lens OL. The position of $I_{3}$ with respect to the objective lens is depicted by c:

(2) $$c=\frac{\left(2u-f\right)f}{2\left(f-u\right)}.$$

The intersection at point $I_{4}$ is regarded as the image of the intersection at point $I_{3}$ through the tube lens TL with the distance (ρ−d) between the tube lens and objective lens, and we have the position of $I_{3}$ with respect to the tube lens as (ρ−d−c). Accordingly, the distance from the focusing point of the beams to the tube lens, *p*, is computed based on the relation of the image and object through the tube lens:

(3) $$p=\frac{\left(\rho -d-c\right)f^{\prime }}{\rho -d-f^{\prime }-c}=\frac{\left(\rho -d-\frac{\left(2u-f\right)f}{2\left(u-f\right)}\right)f^{\prime }}{\rho -d-f^{\prime }-\frac{\left(2u-f\right)f}{2\left(u-f\right)}}.$$

The relation between 2 angles α and β is expressed as

(4) $$\frac{\tan\beta }{\tan\alpha }=\frac{b}{c}=\frac{2\left(f-u\right)}{f}.$$

Similarly, the relation between γ and β is expressed as

(5) $$\frac{\tan\gamma }{\tan\beta }=\frac{\rho -d-c}{p}=\frac{2\left(\rho -d-f^{\prime }\right)\left(u-f\right)-\left(2u-f\right)f}{2\left(u-f\right)f^{\prime }}.$$

Combining (4) and (5), and by using $tan\alpha =\frac{l}{f}$ according to Figure 1, we obtain

(6) $$\tan\gamma =-\frac{2\left(\rho -d-f^{\prime }\right)\left(u-f\right)-\left(2u-f\right)f}{f^{\prime }f}tan\;\alpha .$$

Furthermore, the distance between two beam spots, which are generated from holes at positions 0 and −1 (or +1), is computed as follows:

(7) $$v=\left(p-d\right)\tan\gamma =-\frac{\left[2u\left(d^{2}-\left(\rho -f\right)d+\rho f^{\prime }-ff^{\prime }\right)-2fd^{2}+2f\rho d-2ff^{\prime }\rho -f^{2}d+f^{2}f^{\prime }\right]l}{f^{2}f^{\prime }}.$$

Differentiating both sides, we obtain

(8) $$\Delta v=-\frac{2\left(d^{2}-\left(\rho -f\right)d+\rho f^{\prime }-ff^{\prime }\right)l}{f^{2}f^{\prime }}\Delta u=-\vartheta .\Delta u.$$

The value ϑ is representative of the resolution of images, which indicates the variation rate in beam-dot spacing according to the OL–specimen distance in the situation of the mask with two holes at positions 0 and −1 (or +1). For the situation of the mask with two holes at positions −1 and +1, the resolution is 2ϑ because the distance between two beam spots on the image sensor is 2v. In the experiment, we can precisely control the parameter Δu by shifting the specimen with increments of 50 μm using the micro-positioning system. The value of the parameter Δv can be recorded using image sensor software. The must-do step with the specimen is to find the value ϑ based on experimental data on Δu and Δv. Thereafter, when the specimen was replaced by the target sample, the defocusing distance of target sample Δu can be known according to the value Δv read by image sensor software. From that, the signal will be transmitted to the micro-positioning system to shift the target sample to the focus.

## 3. Experimental Methods

### 3.1. Optical Design for Focus Detection and Fabrication

**Figure 2:**
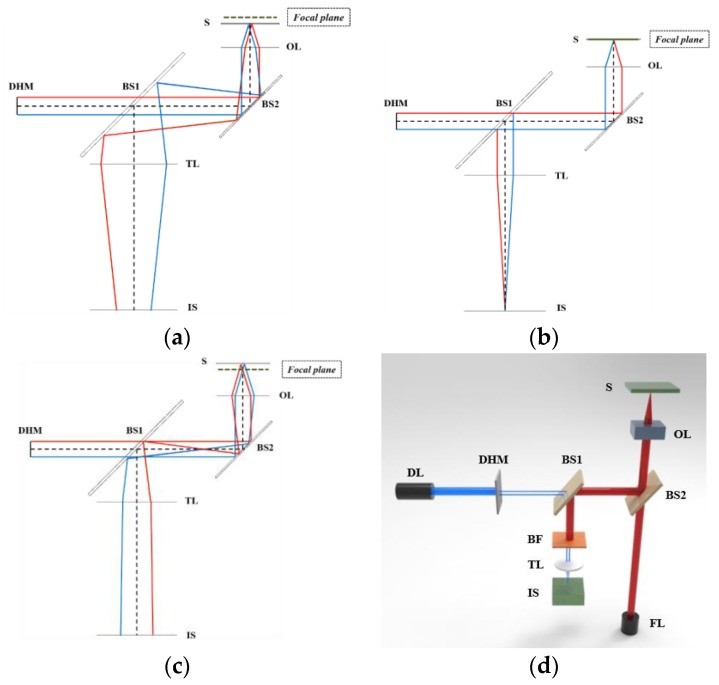
Schematic of the optical system for focus detection and fabrication. (**a**) The optical system when the specimen is located in front of the focal position; (**b**) the system when the specimen is located at the focus; (**c**) the system when the specimen is located behind the focal position; and (**d**) the system when two laser sources operate simultaneously during laser fabrication.

A basic description of the established optical system for rapid, non-destructive, and precise detection of focal position is shown in Figure 2, which shows the configurations of the optical system when the specimen is positioned in front (a), at (b), and behind (c) the focus as well as when the laser fabrication step is carried out (d). The system includes a nanosecond laser (redENERGY G4 fiber laser S-type, SPI Lasers) with a wavelength of 1064 nm for fabrication, a laser diode with a wavelength of 655 nm for focus detection, an objective lens (M Plan Apo NIR 5x, Mitoyo, Japan), a specimen (silicon wafer), a bandpass filter (FB780-10, CWL = 780 nm, FWHM = 10 nm, Thorlabs) that allows the transmission of 655 nm wavelength beams, prevents the transmission of 1064 nm wavelength beams, and allows adjustment of the beam spot on the image sensor by removing the speckle patterns induced by the roughness of the target surface, an achromatic tube lens with a focal length of 100 mm, and two beam splitters. The detection beam is produced from the diode laser (DL). This beam passes through a double-hole mask (DHM) and gets divided into two parallel fractional beams. Parallel beams are directed to the specimen by beam splitter 2 (BS 2) and then reflected and converged to the image sensor (IS) through the bandpass filter (BF), which allows for the transmission of 655 nm wavelength to the tube lens (TL). After the focal point is well detected, the fabrication laser is operated to fabricate the micro-patterns. A high-power fabrication laser (FL) beam with a wavelength of 1064 nm is generated from the nanosecond laser source. This beam is directed to the specimen through the objective lens. The reflection beam from the specimen is divided into 2 fractional beams by BS 2. One fractional beam propagates to the fabrication laser source and the other is prevented from reaching the tube lens and damaging the image sensor during fabrication by the bandpass filter. Both the detection laser and fabrication laser are operated simultaneously to maximize the productivity of fabrication at the focus. The laser diode can be automatically adjusted to guarantee that the spherical aberration effect is minimized and the images are properly achieved on the image sensor.

**Figure 3:**
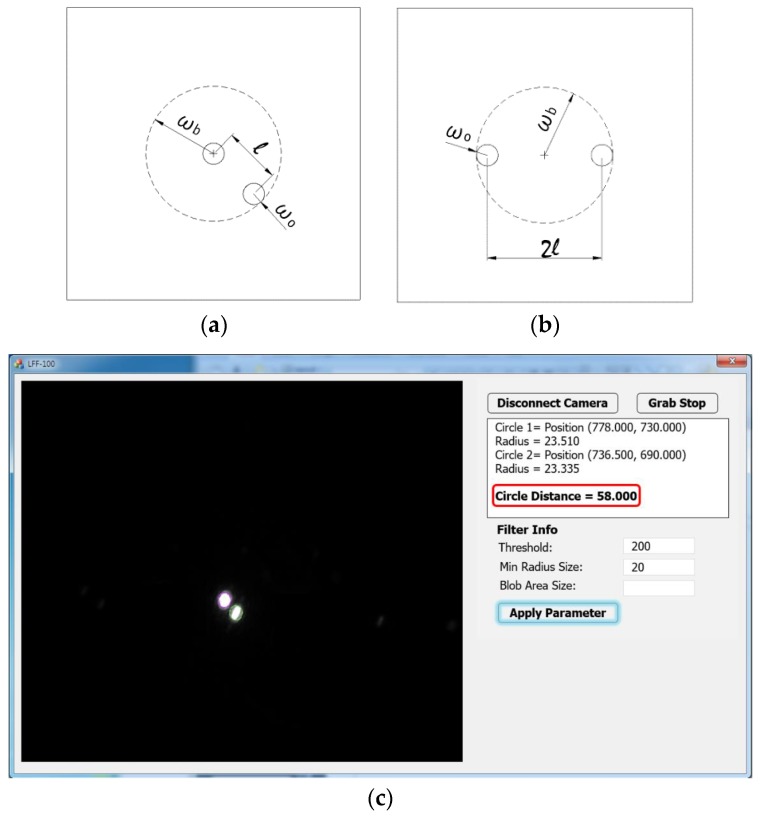
Metal double-hole masks (**a**,**b**) and the control system interface of the image sensor software (**c**).

The metal double-hole masks with two 0.5 mm diameter holes are shown in Figure 3a,b. Figure 3a illustrates the double-hole mask with holes at positions 0 and −1 (or +1), and Figure 3b illustrates the double-hole mask with holes at positions −1 and +1. The resolution of the double-hole mask in Figure 3b is twice that of the double-hole mask in Figure 3a. Therefore, the one in Figure 3b is selected for better observation of beam spots on the image sensor. Moreover, because the beam-spot size is limited and it is required to completely cover the two holes, the mask is selected such that the beam-spot size is $w_{b}=l+w_{o}$, where $w_{b}$ is the beam-dot radius, 2l is the hole spacing, and $w_{o}$ is the hole radius. We employ the image sensor named u-Nova20M (1600 × 1200, 50 fps, Mono, Global Shutter, CMOS) integrated with image feature reading software, which can precisely read the spacing between two beam dots on the image sensor based on the two peak intensities in Figure 3c. Furthermore, a silicon wafer is used as the specimen for the calibration step. The specimen will be replaced by a target sample, which is fabricated after the calibration is completed and all optical elements including the lenses, beam splitter, and double-hole mask are well manufactured in a frame known as an optical box.

### 3.2. Calibration for Focus Detection

**Figure 4:**
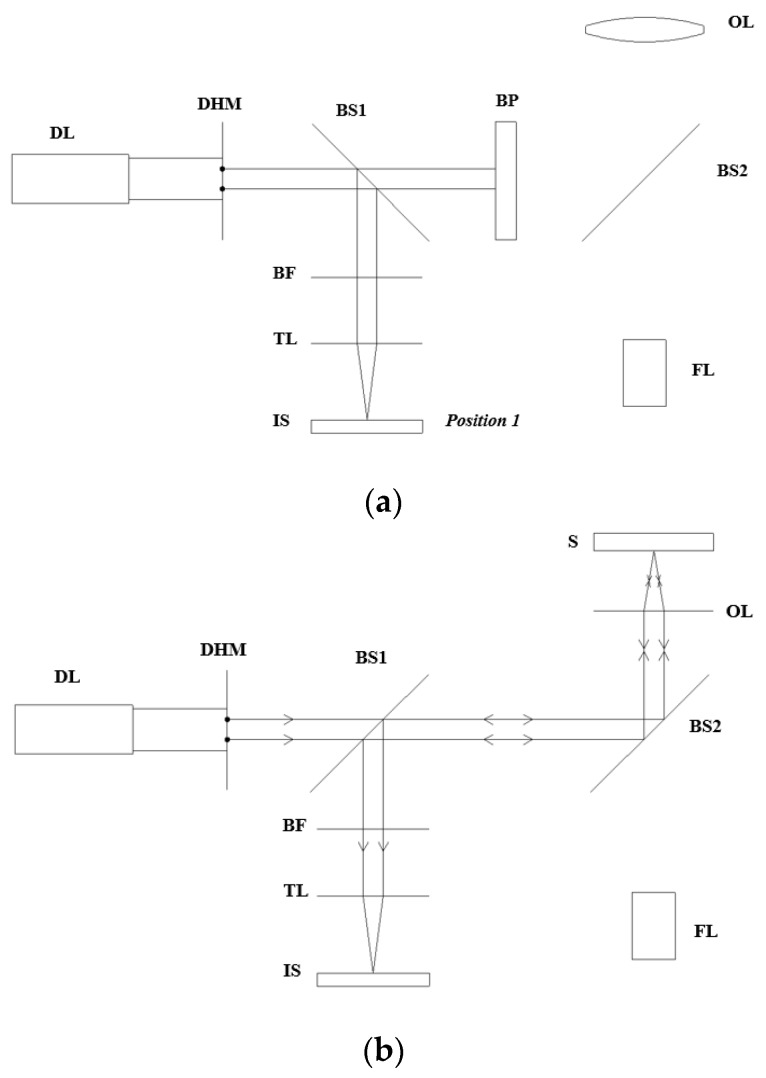

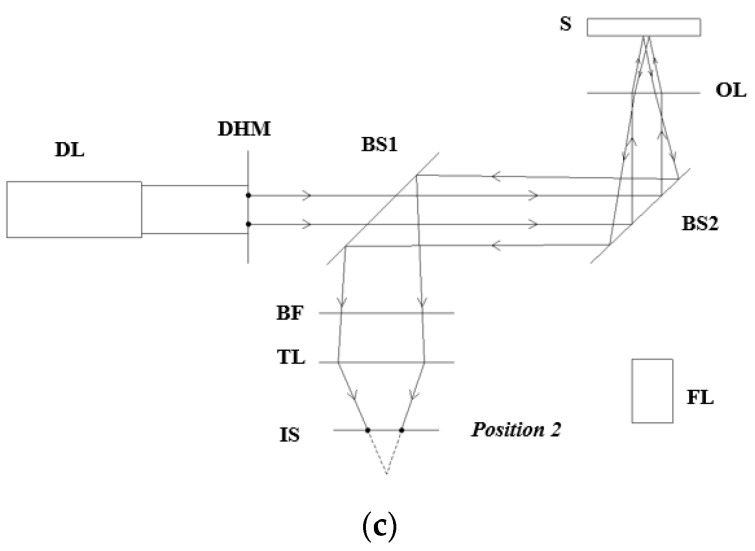
Calibration steps. (**a**) The image sensor is positioned at position 1, at which the overlapping of two beam spots is obtained on the image sensor display while the blocking plate prevents the detection laser from reaching the objective lens; (**b**) The blocking plate is removed and the specimen is located at the focus; (**c**) The image sensor is located at position 2, at which it can detect the beam-spot spacing without interference while the specimen is located at the focus.

The main limitation of the image sensor is the fact that it cannot read the beam spots’ distance when these spots start overlapping each other because of interference between peak intensities. To overcome this limitation, the calibration step is conducted in advance to prevent two beam spots from approaching each other. Furthermore, the calibration is an essential step applied on the specimen to determine the focal point before the specimen is replaced by a target sample for fabrication because it identifies the spacing between two dots on the image sensor when the sample is positioned at the focus. The calibration step is performed based on the optical setup as follows:

- Place the blocking plate to prevent the measurement laser (diode laser) from reaching the objective lens and tilt beam splitter 1 by 45° with respect to the optical axis.
- Move the image sensor along the optical axis until the concentricity of two beam spots is observed by the naked eye in the image sensor display, and record this position of the image sensor at position 1 (Figure 4a).
- Remove the blocking plate and rotate beam splitter 1 by 90° with respect to the initial position to direct the reflected beam from the specimen to the image sensor.
- Move the specimen by increments of 50 μm until the concentricity of beam spots is again observed on the camera; record this position as the focal position (Figure 4b).
- Keep the other optical elements and specimen stable and move the image sensor to the new position rather than position 1 until two beam spots are split completely and the image sensor can read the distance between them; record this distance and the position of the image sensor as position 2. The recorded distance between the two beam spots on the CCD will later indicate the focal position of the target sample (Figure 4c). Whenever the distance between two beam spots on the CCD camera reaches this recorded value, the focal position of the target sample is detected.
- Replace the silicon sample (specimen) with the real target sample on the micro-positioning stage, process the focus detection as aforementioned, and eventually perform the patterning using the fabrication laser.

Owing to this calibration step, we can overcome the limitation of the image sensor and skip the step of pre-measurement of optical-element distances, which is probably the root of error. Furthermore, a laser power filter can also be used to reduce the laser intensity before the high-power laser beam strikes the camera surface to protect it from damage. The fabrication laser is only operated when the sample is at the focus, and it is prevented from reaching the image sensor by the bandpass filter, which only allows the beam of 655 nm wavelength to be transmitted. The detection laser (diode laser) was operated during detection and fabrication to manipulate the sample position at the focus. The spherical aberration effect and any small misalignment can be ignored in this technique without impacting the accuracy of the measurement. In the future, the image sensor software can be connected with the micro-positioning system of the sample to locate the target sample at the focus automatically, according to the measured distance between beam dots. An optical box is manufactured to keep optical elements, including the double-hole mask, beam splitters, objective lens, and tube lens, stable and in the best alignment.

## 4. Experimental Results and Discussion

**Figure 5:**
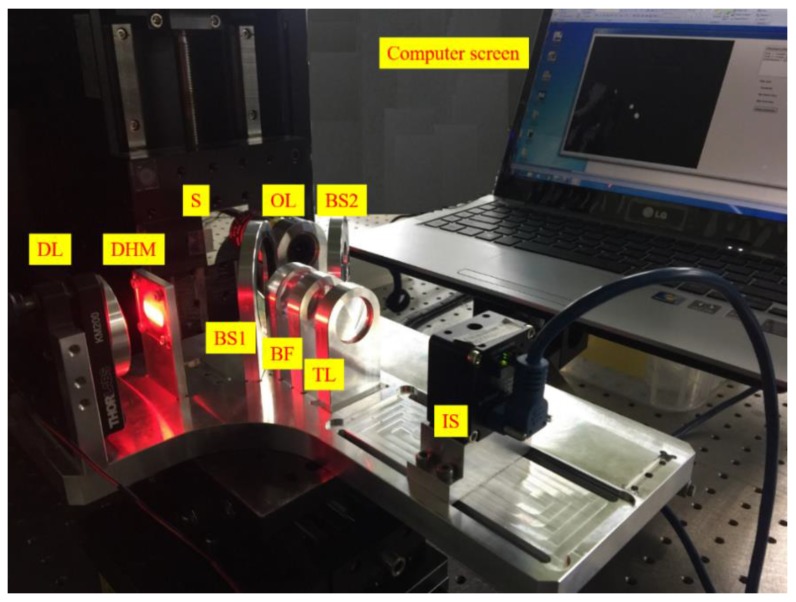
Experimental setup.

The experiment is performed following the optical setup and optical elements mentioned in Section 3.1. A photograph of the experimental system is shown in Figure 5 when the optical elements are well arranged in the optical box. Based on the experimental method and analytical models (Equation (8)), we can examine the resolution of this technique and determine the optimal solution to enhance the resolution. Specifically, the resolution will increase when a small misfit of the focal position can lead to a significant displacement of beam spots on the image sensor. Furthermore, owing to the spherical aberration, the small beam-spot size on the image sensor will also contribute to the increase of resolution of the technique.

**Figure 6:**
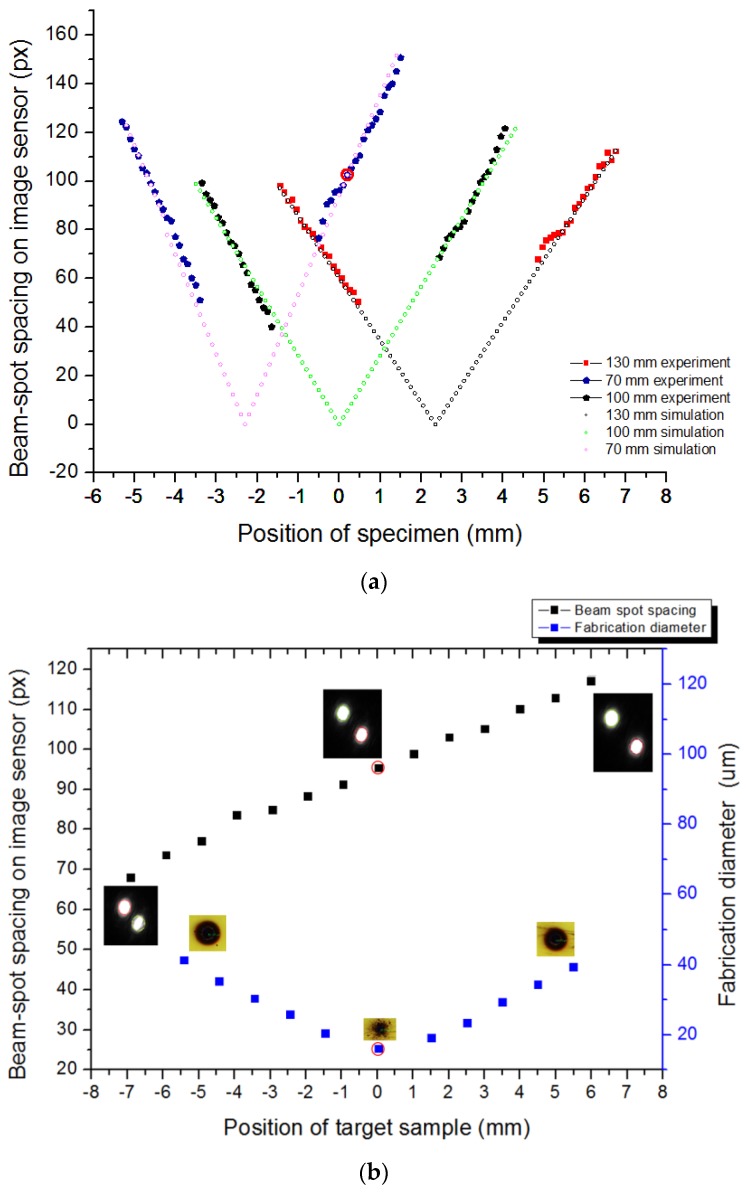
Experimental results of focus detection and fabrication. (**a**) The relation between changes in OL–specimen distance and beam-spot spacing on the image sensor for each position of the image sensor (each color corresponds to a position of the image sensor) with respect to the tube lens; (**b**) Fabrication results and changes in beam-spot spacing on the image sensor when the target sample is located at different positions including the focal position and when the image sensor is positioned 70 mm from the tube lens.

Figure 6a below shows the experimental and simulation results of the calibration step at different positions of the image sensor with respect to the tube lens corresponding to different colors, which result from the linear change in the distance between beam spots on the image sensor as the sample is shifted in increments of 50 μm along the z-direction. The vertical axis indicates the beam-spot spacing, and the horizontal axis indicates the OL–specimen distance. Accordingly, the slopes of the graphs are the values of 2ϑ at different values of d when ρ is fixed. The slope of the line graph increases as the image sensor comes closer to the tube lens, implying that the image resolution increases as the distance between the image sensor and tube lens decreases. Furthermore, it is clearly seen that there is a range at which we could not obtain the beam-dot spacing data on the image sensor, owing to the interference of two beam dots when they approach each other, as mentioned in the calibration method (Section 3.2). This result implies a great unification between the theoretical model and practical performance of our optical design for focus detection. Based on this linear relation, the target sample can be easily located at the focus. In addition, the defocusing distance and direction can also be explored according to the relative position of the side dot compared with the central dot. For a real target sample with a rough surface, the system can be based on this relation to manipulate the sample at the focal position of the laser beam through the change in distance between beam spots on the image sensor. The positions of the image sensor and other optical elements, such as the laser diode, double-hole mask, beam splitters, objective lens, bandpass filter, and tube lens are fixed in a complete optical box for conducting the focus detection and fabrication of any sample. After we conduct the calibration step, the beam-dot image generated from the target sample is captured, and fabrication is performed using the high-power laser.

Figure 6b indicates the fabrication results as well as the changes in beam-spot spacing on the image sensor when the target sample is located at different positions, including the focal position and when the image sensor is positioned 70 mm from the tube lens (the blue line in Figure 6a). The reason why the blue line is chosen is that we can detect the focal position (the point marked by the red circle) at which two beam dots are not overlapped when the image sensor is placed at this position (70 mm from the tube lens). The left vertical axis indicates the beam-spot distance on the image sensor while the right vertical axis illustrates the drilled hole diameter. The horizontal axis indicates the position of the target sample along the z-axis. The micro-holes drilled by the fabrication laser are observed by microscopy (Microscope-Nikon LV150, Nikon, Tochigi, Japan). The images of the micro holes and beam spots in the middle of line graphs are obtained when the specimen is positioned at the focus, which is marked by red circles. The images of micro-holes and beam spots on the other sides are captured when the specimen is located at out-focusing positions around the focal point. From Figure 6b, it is clear that the micro-hole fabricated at the focus has the smallest diameter, because it involved the maximum laser intensity among all micro-holes fabricated at defocal positions. This implies that an accurate focusing condition can produce high-quality nano-patterns on the target sample. During the fabrication, based on the relation between distances, the target sample position is always automatically manipulated at the focus. The entire process occurs quickly and effectively. It is suitable for massively producing nano-patterns on various sample surfaces.

We will analyze the detection errors, offer possible solutions to reduce these errors, and describe the strengths and weaknesses of the technique. First, the detection error probably originates from several sources, such as the speckle effect due to reflection on the rough target surface, the misalignment of optical elements, and the identification of overlapping of two laser dots using the naked eye. To reduce these errors, a laser diode (detection laser) is automatically adjusted to eliminate speckle patterns. Furthermore, because the image sensor software can read the beam-dot spacing based on intensity peaks, the distorted beam shape caused by sample roughness and the small misalignment can be ignored. In addition, the linearization of the relation between changes in objective lens–specimen distance and beam-dot spacing can partly reduce the error caused by the identification of overlapping.

The resolution of detection can increase when we select an objective lens with smaller focal length and a double-hole mask with larger hole spacing. The resolution also increases when the distance between the image sensor and tube lens decreases in the range of the micro-positioning stage according to the resolution formula of 2ϑ. Furthermore, according to this formula, the image resolution also depends on the distances between optical elements as well as the focal length of single lens. Theoretically, the resolution will be higher for a smaller focal length of the tube lens based on $\vartheta =\left(\frac{2\left(d^{2}-\left(\rho -f\right)d\right)l}{f^{2}f^{\prime }}+\frac{2\left(\rho -f\right)l}{f^{2}}\right)$. As one can see the experimental result as line graphs, the resolution makes the detection more observable when the small shift in OL–specimen distance leads to a large change in beam-spot spacing.

In terms of technical advantages, the optical design enables in-situ real-time focus detection and laser fabrication simultaneously. The technique can be applied for various target samples. By using the bandpass filter, two laser sources can be operated simultaneously without damaging the image sensor. The bandpass filter also contributes to polishing the beam-spot images. The detection is performed in real time by positioning the sample at the focus immediately when fabrication is performed, rapidly, with automatic control based on recorded data from the image sensor, reproducibly, owing to the capability of mass production of the patterned sample without a reconstruction of the system, and accurately, with the support of an advanced image sensor, which can scan the beam-spot spacing on the basis of intensity peaks. The optical box is simple, compact, light, and cheap. The calibration may also be performed comfortably using any specimen with a polished surface. However, the usage of two laser sources may be inconvenient for engineers. Nevertheless, this weakness does not narrow the broad application potential of this method in academic research in general, and laser processing in particular.

## 5. Conclusions

The proposed technique is a breakthrough in the in-situ real-time detection of focal position, as it can scan the focal position rapidly and simultaneously with the fabrication process using a double-hole mask and two laser sources. Based on the advantages of the double-hole mask, the analytical model for the expression of beam-spot distance and size with respect to the defocusing shift offers a reliable prediction for experimental data and gives a stringent focal condition for laser micro-machining. The laser band filter prevents the fabrication laser from destroying the image sensor and adjusts the beam-spot images, removing speckle patterns generated by rough morphology on the target surface. This contributes to an increase in the accuracy of data recording. Furthermore, according to the theoretical calculation, the optimized condition of resolution for detecting the focal point was determined, and thus, the productivity of the detection process was increased with high reproducibility. With the capability to create two sampled beam clusters with low intensity, this technique can lead to a new generation of diffractive detection systems dealing with focus-finding problems using high-intensity lasers. Finally, the fact that the target sample is positioned at the focus immediately as the fabrication is performed, demonstrates the high speed of the technique. The experimental technique is integrated with analytical models to construct an algorithm for an auto-focusing program, which is expected to be applied widely in the industry.
